# Analysis of bone mass and its relationship with body composition in school-aged children and adolescents based on stage of puberty and site specificity: A retrospective case–control study

**DOI:** 10.1097/MD.0000000000014005

**Published:** 2019-02-22

**Authors:** Cui Song, Min Zhu, Rongfei Zheng, Yujuan Hu, Rong Li, Gaohui Zhu, Long Chen, Feng Xiong

**Affiliations:** a Department of Endocrine and Genetic Metabolism Disease, Children's Hospital of Chongqing Medical University, Ministry of Education Key Laboratory of Child Development and Disorders; b China International Science and Technology Cooperation Base of Child Development and Critical Disorders, Chongqing Key Laboratory of Pediatrics, Chongqing; c Endocrinology Departments, Shenzhen Children's Hospital, Shenzhen, P.R. China.

**Keywords:** adolescent, body composition, bone mass, school-aged children, site specificity

## Abstract

The aim of this study was to better understand the relationship of bone mass with body composition based on different stages of puberty and to illuminate the contribution of site-specific fat mass and lean mass (FM and LM) compared with bone mass in school-aged children and adolescents in Chongqing, China.

A total of 1179 healthy subjects of both sexes were recruited. Bone mineral content (BMC), bone mineral density (BMD), bone area, and both FM and LM were measured by dual-energy X-ray absorptiometry (DXA). The fat mass and lean mass indexes (FMI and LMI, respectively) were calculated as the FM (kg) and LM (kg) divided by the height in meters squared, respectively.

Most of the bone mass indicators were significantly higher for postpubertal boys than for girls at the same stage (*P* < .001). The proportion of subjects with normal bone mass increased, while the proportion of subjects with osteopenia and osteoporosis decreased with increased body weight regardless of gender and puberty stage (*P* < .01). FM and LM were significantly positively related to bone mass regardless of gender and puberty stage (*P* < .0001). FMI and LMI were significantly positively related to bone mass in most conditions (*P* < .05 and *P* < .0001, respectively). Four components of the FM and LM were linearly and significantly associated with BMD and BMC for TB and TBHL. Among them, the head fat mass and head lean mass showed the greatest statistical contribution.

In the process of assessing bone status, we recommend measuring fat and lean masses, including the fat and lean masses of the head.

## 1 Introduction

Osteoporosis is characterized by low bone mass and high fracture risk and has been recognized as a worldwide public health challenge. Osteoporotic fractures are associated with long-term negative health effects, including increased mortality and economic burden. These effects could be mitigated or avoided by improving the ways in which bone health status can be monitored using bone mineral density (BMD), a well-known indicator.^[1]^ School age and adolescence are critical periods for the growth and development of the body, including the bones. Half of the peak bone mass (PBM) is attained during this time, and adequate attainment during this period is beneficial for reducing fracture risk later in life.^[2,3]^ Although 60% of PBM is genetically determined, there are many other influential factors, including dietary intake of calcium and vitamin D, use of medications, the presence of obesity, physical activity, and certain chronic diseases (type 1 or type 2 diabetes, inflammatory bowel disease, cystic fibrosis).^[3,4]^ If adequate PBM is not achieved due to any of these factors, the patient is at risk for later osteoporosis and fracture.^[2]^ However, precise evaluation of BMD status during childhood and adolescence may allow individuals with low BMD to undergo positive interventions, making it important both for individuals and for the society as a whole.

Dual-energy X-ray absorptiometry (DXA) has been recommended by the International Society for Clinical Densitometry (ISCD) as a standard method of measuring BMD and body mineral content (BMC).^[5]^ In addition to BMD and BMC, DXA is also capable of measuring fat mass (FM) and lean mass (LM). This measurement tool has the following advantages: ease of use, high efficiency, low radiation exposure, and strong correlation with established methods.^[6–10]^ However, some circumstances may lead to inaccurate BMD results using DXA. A previous study indicated that precision errors may occur when DXA is used in individuals who are obese, likely due to abdominal wall thickness and beam hardening effects.^[11]^

The 3 major metrics for body composition are BMC, FM, and LM, all of which can be easily measured by DXA.^[12]^ Among them, FM and LM are the 2 major components of body mass index (BMI: kg/m^2^). In addition to FM and LM, fat mass index (FMI; fat mass/height^2^) and lean mass index (LMI; lean mass/height^2^) are other indicators of body composition. Current studies have yielded conflicting findings with respect to the effects of these body composition indicators (and particularly of the effect of fat) on fracture. Most existing studies support the concept that fracture risk is lower at the proximal femur and the spinal vertebrae in individuals with obesity;^[13]^ conversely, the risk of some nonspinal fractures at sites such as the proximal humerus, ankle, and upper leg is relatively higher.^[14,15]^ With respect to the LM that is used to maintain nutrition and function of body, it represents the protein reserve of the used by the body to a great extent during catabolic periods.^[16]^ The development of reduced LM deposition in childhood may have implications for the risk of diseases such as osteoporosis and related disorders in adulthood.^[17]^ Prevention of osteoporosis may be achieved by both the precise measurement of BMD and an enhanced understanding of the relationship between body composition and bone health/fracture risk, including how these outcomes are differently impacted before and after puberty.

Although previous studies have reported the effects of FM or LM on BMD, few studies have been conducted to evaluate their effects on skeletal outcomes in the same population using multiple indices, especially in children and adolescents. Differences in this association before and after puberty have also not been well examined, and reports have been particularly rare with respect to site-specific FM or LM and its relationship to bone outcomes at these sites.

In this study, children and adolescents aged 6 to 19 years and from Chongqing, China were enrolled. The overall objective was to evaluate each subject's bone health and analyze the relationship between skeletal outcomes and FM and LM, specifically how FM and LM may be related to bone mass measurements using DXA. These data may provide researchers and clinicians with useful information and facilitate improved evaluation of overall bone status in children and adolescents.

## 2 Patients and methods

### 2.1 Subjects

A total of 1179 healthy school-age children and adolescents aged 6 to 19 years (581 boys, 598 girls) were recruited from 6 local schools in Chongqing in southwest of China. All subjects enrolled in the study were of Chinese ethnicity and of good health. Subjects completed both a screening questionnaire and a physical examination by trained pediatricians for the purpose of obtaining demographic information and assessing health status, respectively. The exclusion criteria included: age of < 6 years or > 19 years; height not within the range of the 3rd to 97th percentiles; a history of medication use or disease affecting bone growth and metabolism; a history of fracture; and the inability to lie flat and hold the necessary position for the time required to complete the scanning procedure. Written informed consent from each subject as well as their parents or guardians was obtained for all participants. Ethics approvals were granted by the ethics committee of the Children's Hospital of Chongqing Medical University.

### 2.2 Anthropometric measurements

Anthropometric data were collected while the subjects were in light clothing without shoes. Height and weight were measured by stadiometer and platform digital scales with a precision of 0.1 cm and 0.1 kg, respectively. Height and weight were measured twice and the mean was taken for the final results. Waist circumference (WC) was measured in centimeters to the nearest 0.1 cm twice using inextensible anthropometric tape positioned parallel to the floor and it was measured midway between the lowest border of the rib cage and the upper border of the iliac crest, at the end of normal expiration.

### 2.3 Bone mass and body composition measurements

Measurements for BMC (g), BMD (g/cm^2^), and bone area (BA) (cm^2^) for the total body (TB) and total body less head (TBLH) and FM (kg), LM (kg) by using Hologic Discovery (A, W, and Wi) fan-beam densitometers (Hologic, Bedford, MA). The coefficient of variation (CV %) was used as the quality control procedure. The CVs of A, W, and Wi were 0.471%, 0.302%, and 0.358%, respectively. All DXA values were analyzed using Hologic Apex software Version 4.0 following the manufacturer's guidelines. The DXA machine was calibrated every morning; all participants were asked to take off their coats when scanning so as not to interfere with the test results. All DXA measurements were performed by a well-trained and qualified operator for the duration of the study.

The training and testing procedures were as follows. First, the operator was trained with training materials that included the ISCD's official technician hands-on training materials and the manufacturer's handbook. The operator was evaluated according to the precision achieved with scans of the lumbar spine, hip trochanter, and femoral neck in 15 participants (each site was scanned 3 times for the calculation). Participants had to leave the scanner between scans to reposition themselves. The operator passed the precision quality control required by ISCD, with actual CV percentages of 0.59% for the lumbar spine (less than the ISCD 1.9% threshold), 1.62% for the hip trochanter (less than 1.8%), and 2.05% for the femoral neck (less than 2.5%). The lean and fat tissue composition variables were calculated from the DXA total body scans as follows: LMI [LM (kg)/height^2^] (kg/m^2^), FMI [FM (kg)/height^2^] (kg/m^2^).

### 2.4 Statistical analysis

The descriptive data include the bone mass values in this study are presented as the mean values ± standard deviation (SD). Data were analyzed by Student *t* test or one-way ANOVA analysis for continuous variables, and the *χ*^2^ test was applied for the categorical variables. The Pearson correlation test was used for detecting the correlations between fat and lean mass and bone mass, and their potential contributions to the skeletal outcomes were further analyzed via the multiple linear regression analysis. *P* values < .05 were considered to be statistically significant. Statistical analyses were performed using SAS software (version 9.4 SAS Institute Inc, Cary, NC) for Windows. The statistical analyses included in this study were completed under the guidance of experts in the Statistics Department.

## 3 Results

Table 1 presents the descriptive characteristics and measurements of the study participants. The mean BMC, BMD, and BA of the total body were 1380.884 ± 544.386 g, 0.874 ± 0.147 g/cm^2^, and 1526.427 ± 344.661 cm^2^ for boys and 1336.379 ± 422.326 g, 0.880 ± 0.144 g/cm^2^, and 1481.268 ± 245.658 cm^2^ for girls, respectively. The TB BMC, TBHL BMC, TBHL BMD, BA, and TBHL BA were significantly higher for boys than for girls at the same postpuberty stage (*P* < .001).

**Table 1:**
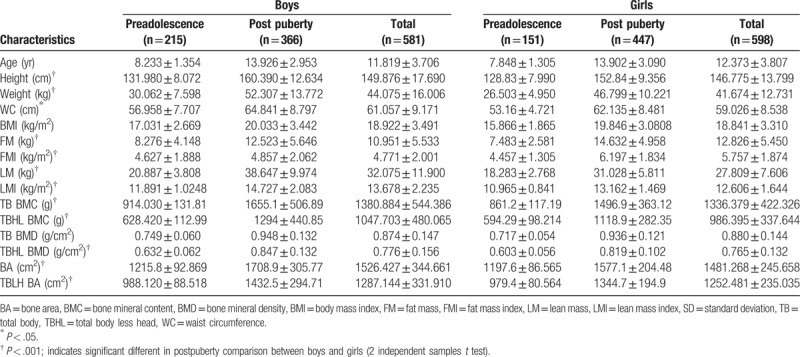
Characteristics and measurements of the subjects (mean ± SD).

Tables 2 and 3 show that the proportion of subjects with normal bone mass increased with increases in body weight, while that of subjects with osteopenia and osteoporosis decreased, regardless of gender and puberty stage (*P* < .01). Tables 4 to 7 show that FM and LM were significantly positively related to BMC, BMD, and BA for TB and TBHL regardless of gender and puberty stage (*P* < .0001). FMI was significantly positively related to bone mass for most conditions (*P* < .05), but it was not associated with TB BMD or TBHL BMD for postpuberty boys or to BA for preadolescent girls. LMI was also positively correlated with bone mass in most cases except for BA and TBHL BA in preadolescent girls.

**Table 2:**
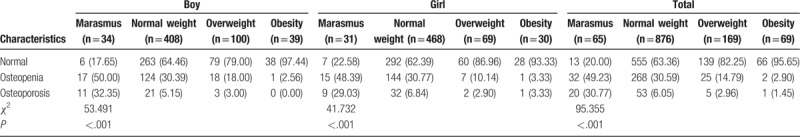
Bone health status with different body weights.

**Table 3:**
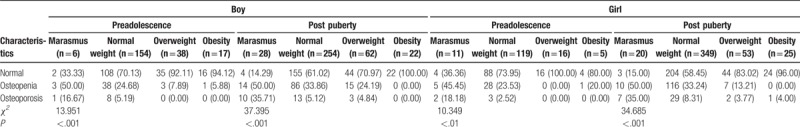
Bone health status with different body weights for boys and girls at different stages of puberty.

**Table 4:**
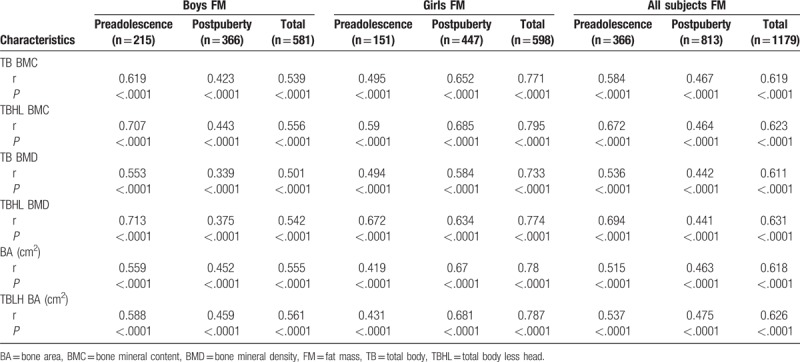
Correlation analysis between FM and bone mass for boys and girls at different stages of puberty.

**Table 5:**
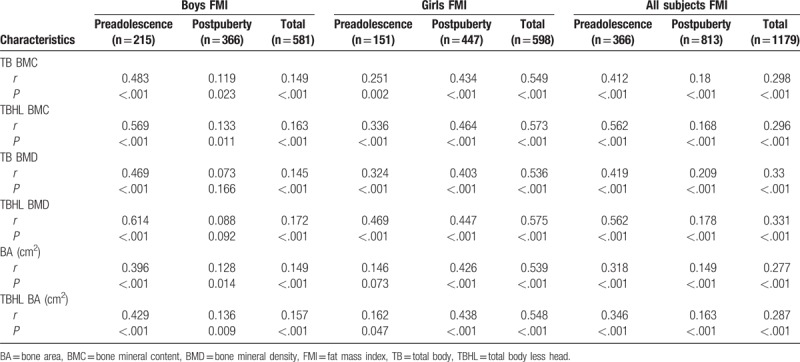
Correlation analysis between FMI and bone mass for boys and girls at different stages of puberty.

**Table 6:**
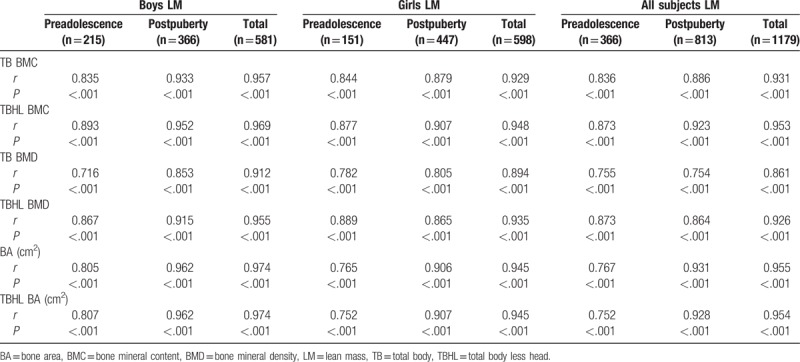
Correlation analysis between LM and bone mass for boys and girls at different stages of puberty.

**Table 7:**
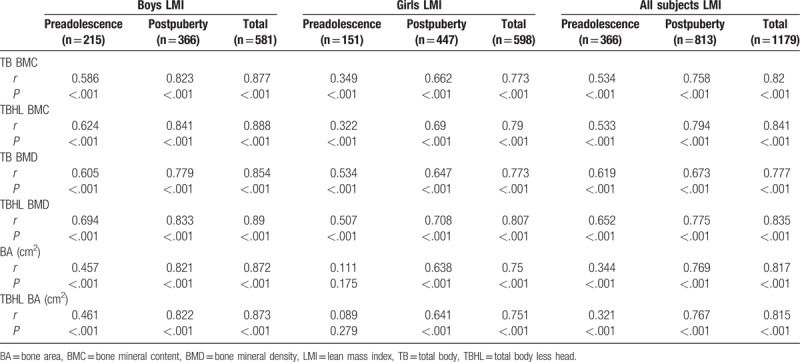
Correlation analysis between LMI and bone mass for boys and girls at different stages of puberty.

Table 8 presents the multivariate regression analysis of regional fat and lean mass compared with bone mass, which showed that head fat mass and fat tissue in the largest visceral fat region were significantly negatively correlated with BMC and BMD for TB and TBHL in most cases (*P* < .05), while head lean and gynoid lean mass were both positively correlated with BMC and BMD for TB and TBHL (*P* < .05). Furthermore, the head fat and head lean masses showed the greatest statistical contribution to BMC and BMD for TB and TBHL.

**Table 8:**
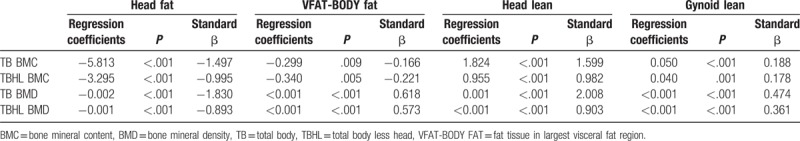
Multivariate regression analysis of regional fat and lean mass against bone mass.

## 4 Discussion

In this study, we evaluated the bone mass of school-aged children and adolescents in Chongqing, China. The average levels of BMC and/or BMD (TB or TBHL) of subjects in this study were lower than those in comparable populations in the United States, Thailand, or Korea, but higher than those shown in a study in India.^[18–21]^ In comparison with children and adolescents of the same ethnicity living in other areas of China, the TBHL BMD was lower in Guangzhou and Jilin than in Chongqing, but it was higher in Chongqing than in Beijing, Shanghai, and Ningxia.^[22,23]^ Many factors influence bone mineral accumulation during childhood and adolescent development, among which heredity is a definitive factor. Different genetic backgrounds should partially explain the differences in bone mass between children and adolescents in China and those in other countries. Other influential factors include diet, physical activity, birth weight, endocrine status, and sporadic risk factors such as cigarette smoking, alcohol, and coffee over-consumption along with insufficient exposure to sunlight. Children and adolescents of the same race may have different bone masses and compositions due to this substantial set of other factors. Differences in bone mass are also evident according to sex and stage of adolescence. Our data showed that both the BMC and BA for TB and TBHL and BMD for TBHL of postpubertal boys were significantly higher than that of girls at the same stage of life, which is consistent with the results of other studies.^[24,25]^ Generally, bone mineral mass consolidation continues reaching plateaus for more than 1 year after the end of the growth spurt during puberty, with nearly 60% of the whole-body BMC of adulthood being achieved by the time of the peak height velocity.^[24,26,27]^ During and after puberty, testosterone, and estrogen levels significantly increase in boys and girls, leading to increased accumulation of LM and FM.^[28,29]^ Boys tend to gain more muscle than fat, while girls gain more fat than muscle. Both LM and FM are positively associated with bone mass; however, the former may be more relevant.^[23]^ This may partially account for the differences in the rate of bone mass increase between boys and girls after puberty. Furthermore, as LM and BMC are regulated by similar hormonal mechanisms,^[30]^ they show common patterns of change. One study showed that the age-related increase in LM was steeper in boys than in girls for the same percentiles after puberty.^[23]^

Our data showed that subjects had different incidences of osteopenia and osteoporosis according to weight. Indeed, the proportion of normal bone mass increased with increases in body weight, while the proportion of bone sites showing osteopenia and osteoporosis increased with decreases in body weight; the difference was statistically significant and consistent with existing research.^[31,32]^ Some studies on different sexes and menopausal status (age for men) have found that underweight individuals are usually at higher risk of low BMD than are normal-weight individuals at the same menopausal stage or age group for men. However, these results did not show a statistically significant difference on the basis of sex or physiologic status (age for men).^[33,34]^ Furthermore, differences in the incidence of osteopenia and osteoporosis in individuals with different weights at different pubertal stages have been rarely reported. Our data showed that regardless of sex or adolescent status, the incidence of both osteopenia and osteoporosis was significantly higher in low-weight individuals. Although using DXA to detect BMD in obese people may lead to precision errors due to abdominal thickness and beam hardening effects, other quantitative imaging methods support the concept that individuals with obesity generally have higher BMD, as demonstrated in a study using high-resolution peripheral quantitative computed tomography.^[35]^ Another study used ultrasound to show that calcaneus bone stiffness is greater in individuals with obesity.^[36]^ Explanations have been proposed for the association between body weight and BMD; notably, it has been hypothesized that a higher body weight imposes a greater mechanical load on the bone, with a compensatory increase in bone mass to accommodate this load.^[37]^

Body composition primarily consists of FM and LM, and many studies have analyzed the associations between body composition and BMD, especially in adults. However, the effect of some of these components on BMD is not consistent across different studies in different subjects. Some studies report that FM is the most significant positive predictor of BMD in postmenopausal women,^[38,39]^ while other studies have reported that FM appeared to contribute negatively to BMD in younger men.^[40]^ The correlation between body composition and BMD may even differ within the same study owing to sex and menopausal status (age for men) variances. Cui reported that only LM had a significant positive correlation with BMD at all sites in premenopausal women. In postmenopausal women, FM was significantly positively correlated with BMD at all sites except for Ward's triangle. In younger men, LM made a significant positive contribution to BMD at all sites, whereas FM appeared to contribute negatively to BMD at all sites except the calcaneus. In older men, LM made a significant positive contribution to the BMD at all sites; FM also made a significant positive contribution to the BMD at the forearm and calcaneus.^[40]^ To the best of our knowledge, few studies have reported the effects of FM or LM on bone mass in children and adolescents using multiple indicators and considering both sex and adolescent status. Our study examined the relationships between bone measurements (i.e., BMC, BMD, and BA for both TB and TBHL) and FM, FMI, LM, and LMI, respectively, for different sexes and stages of puberty. The results showed that regardless of sex and pubertal status, FM and LM were significantly positively related to all the bone mass indicators. While FMI was significantly positively related to bone mass in most cases, it was not related to either TB BMD and TBHL BMD in postpubertal boys or to BA in preadolescent girls. As for LMI, it was also positively correlated with bone mass in most cases except for BA and TBHL BA in preadolescent girls.

Fat is one of the major components of the body, and it affects bone health through endocrine pathways. Osteoblast and osteoclast cell number and activity have been shown to be influenced by the following adipocyte-related endocrine factors: circulating leptin, which acts on bone cells directly to increase bone formation,^[41]^ but which may also inhibit bone formation when acting through the hypothalamus due to increased activation of the sympathetic nervous system;^[42]^ adiponectin, which is secreted in inverse proportion to fat mass; in humans, circulating adiponectin levels are inversely related to BMD,^[43]^ and animal experiments suggest that adiponectin may also signal through the central nervous system to regulate bone turnover through autonomic innervation;^[44]^ and estrogen, with high fat mass being associated with higher levels of circulating estradiol, likely leading to positive effects on bone mass.^[45]^ LM, another major physical component, was also positively related to bone mass in our study, which is consistent with some existing studies.^[23]^ Although the association between LM and bone mass has received more attention, a few studies have reported on the association between LM and bone mass in children and adolescents, especially with respect to the effect of different stages of puberty. The exact mechanism by which LM affects bone mass remains unknown. However, Xiang reported that LM should be considered a mediator in the relationship between physical activity and BMD in women after menopause.^[46]^ Greater LM strength has been shown to lead to better physical work capacity, which may be one reason for the positive relationship between the LM and bone mass. Other mechanisms for the relationship between the LM and bone status include mechanical stimuli, hormone levels (including sex hormones and hormones that regulate metabolism), and modulation of the expression of local factors, including proinflammatory cytokines (e.g., interleukin-6, tumor necrosis factor-alpha).^[47]^ In addition, reduction of LM in childhood may increase the risk of disease affecting bone outcomes in adulthood,^[17]^ possibly providing a partial explanation of the association between LM and bone mass.

Our data showed that body weight is significantly related to bone health. In addition, the 2 major body components of body weight were also closely related to bone outcomes, regardless of gender, and adolescent status. However, previous studies have suggested that the association between fat and bone and related fracture outcomes is more complicated and potentially depends on the distribution of fat on the body. For example, although fat mass is positively associated with trabecular microarchitecture, both marrow fat and visceral fat have a negative effect on bone mass.^[48,49]^ As for the impact of fracture, fat plays a different role depending on the site. Fat in the area of the lateral hip may help dissipate impacts from falls and reduce hip fracture risk;^[50]^ conversely, intramuscular fat may lead to high fracture risk by impairing muscle function.^[51]^ Current studies have begun to realize the importance of the association between the regional distribution of FM and/or LM and skeletal outcomes. However, to our knowledge, very few studies have clearly stated the impact of site-specific FM and/or LM on bone health in children and adolescents or illuminated the mechanism of this impact. Our study analyzed the potential impact of site-specific fat and lean on bone mass and found that four components of the FM and LM were linearly and significantly related to BMD and BMC for TB and TBHL. Among them, the site-specific FM negatively influenced bone outcomes in most conditions, while regional LM had positive impacts on bone mass. Interestingly, the bone-mass impact of the head fat and head lean masses always ranked in the top 2 in terms of statistically significant influence. Wang reported that head fat mass in the obese was associated with classic obesity markers such as BMI, WC, hip circumference, visceral index, and basal metabolism in both genders and revealed that head fat might be a predictor for metabolic abnormalities.^[52]^ Marwaha et al^[53]^ reported that regional LM, including LM of the arms, legs, and trunk, was positively correlated with BMC at all sites. Our data showed that head lean and gynoid lean masses were positively correlated with both BMD and BMC for TB and TBHL. Multiple linear regression parameters showed that head LM had a greater impact on BMC and BMD than did gynoid LM. Accordingly, we contend that site-specific fat and lean measurements are promising in terms of bone health assessment, especially the measurement of head LM and FM, which may have important clinical value even though the head is not the main area of fat and lean mass accumulation.

However, our study has a limitation. Although we enrolled as many participants as possible, this was a single-center study. We are preparing a multicenter study to further investigate the role of site-specific fat and lean measurements in children.

## 5 Conclusions

In summary, our data showed that after puberty, boys had higher bone mass than girls. Body weight was closely related to bone health, and the 2 major components of body weight were closely related to bone mass, irrespective of sex and stage of puberty. The most interesting and important finding of our study is that, in addition to the total fat and lean mass, the site-specific determination of regional fat and lean mass, especially head fat mass and head lean mass, has promising clinical value for assessing bone health.

## Acknowledgments

The authors thank the participants and collaborators who contributed to the study here.

## Author contributions

FX conceived and designed the whole study; CS and MZ were responsible for data acquisition and analysis, and they were major contributors in manuscript draft; RZ and YH performed statistical analysis; RL, GZ, and LC made substantial contributions to interpretation of data and participated sufficiently in the work to take public responsibility for appropriate portions of the content; FX revised the manuscript critically for important intellectual content. All authors have read and concurred with the final version of this manuscript.

**Conceptualization:** Rong Li, Long Chen, Feng Xiong.

**Data curation:** Cui Song, Min Zhu, Yujuan Hu.

**Funding acquisition:** Feng Xiong.

**Investigation:** Cui Song, Rongfei Zheng, Yujuan Hu.

**Resources:** Yujuan Hu, Rong Li.

**Software:** Rongfei Zheng, Yujuan Hu, Rong Li.

**Supervision:** Gaohui Zhu, Long Chen, Feng Xiong.

**Validation:** Rongfei Zheng, Rong Li, Gaohui Zhu.

**Writing – original draft:** Cui Song, Min Zhu.

**Writing – review & editing:** Gaohui Zhu, Long Chen.
